# 1.2 million kids and counting—Mobile science laboratories drive student interest in STEM

**DOI:** 10.1371/journal.pbio.2001692

**Published:** 2017-05-16

**Authors:** Amanda L. Jones, Mary K. Stapleton

**Affiliations:** 1 Science Education Department, Seattle Children’s Research Institute, Seattle, Washington, United States of America; 2 Bioscience Education and Outreach, Towson University Center for STEM Excellence, Baltimore, Maryland, United States of America

## Abstract

In today’s increasingly technological society, a workforce proficient in science, technology, engineering, and mathematics (STEM) skills is essential. Research has shown that active engagement by K–12 students in hands-on science activities that use authentic science tools promotes student learning and retention. Mobile laboratory programs provide this type of learning in schools and communities across the United States and internationally. Many programs are members of the Mobile Lab Coalition (MLC), a nonprofit organization of mobile and other laboratory-based education programs built on scientist and educator collaborations. A recent survey of the member programs revealed that they provide an impressive variety of programming and have collectively served over 1.2 million students across the US.

As technology and knowledge about the world continues to grow, a workforce proficient in science, technology, engineering, and mathematics (STEM) skills is essential for ensuring continued economic growth, the ability to invent and manufacture better products, improving healthcare, and protecting the environment and national security [1–4]. Nine of the ten fastest growing occupations in the US now require significant math or science training, and employers in both traditional and nontraditional STEM fields are seeking workers who are already equipped with STEM knowledge [5–8]. Furthermore, in our increasingly technological and scientific society, an understanding of the nature of science and scientific inquiry is critical for all, not just those in the STEM workforce [9].

While some progress in science education has been made in the US, according to the 2015 National Assessment of Educational Progress (NAEP)—known as “The Nation’s Report Card”—only one third of students have the skills they will need to be adequately prepared for college-level science classes and for a career in STEM [10].

The members of the MLC—a nonprofit organization of mobile and other laboratory-based education programs built on scientist and educator collaborations—have long been aware of gaps and challenges in science education such as those identified by the NAEP. The mission of the MLC is to address the nation’s science education challenges by supporting member programs as they provide equity of access to authentic hands-on, inquiry-based contemporary science education for K–12 students, educators, and communities. Each member program of the MLC operates independently and has their own specific objectives that are defined by their affiliation, funding, and level of program maturity. MLC members include academic institutions, foundations, and nonprofit organizations from across the US, as well as two international locations.

There is a substantial body of research demonstrating that active engagement with hands-on learning that includes authentic scientific tools is the most effective way for students to learn and retain science knowledge [11–17]. Integrated laboratory experiences have the potential to expand student learning beyond the simplified instruction presented in lectures and readings, thereby allowing students to master complex science topics [18,19]. Recently released national science standards in the US, which were developed with this body of research in mind, emphasize student engagement in the same scientific and engineering practices used by scientists [9]. Not surprisingly, students with more exposure to science scored better on the NAEP than students with less exposure. Students in eighth grade who participated in hands-on activities or investigations in science class every day or almost every day, as reported by their teachers, scored 12 points higher than students who never or hardly ever engaged in these activities [10]. Students who have teachers with access to scientific tools for teaching science also scored higher. Eighth grade students whose teachers reported the highest level of access to these tools scored 16 points higher than students whose teachers reported no access. For students in 12th grade, the impact of access to scientific tools was even more pronounced, with students who reported having access scoring 37 points higher than students without access [10].

Despite the clear importance of hands-on experiences with scientific tools, many schools lack access to equipment, resources, and trained personnel [10,18,20–22]. Mobile labs can help to fill the gaps and increase equity of access for all students [23–25].

Mobile labs first became popular in the late 1990s with the launch of the Boston University Medical School (Boston, Massachusetts) MobileLab in 1998, followed soon after by programs at the University of North Carolina at Chapel Hill (Chapel Hill, North Carolina); CURE, “the bioscience cluster of Connecticut” (New Haven, Connecticut); MdBio Foundation (Rockville, Maryland); and the J. Craig Venter Institute (Rockville, Maryland). In 2005, these programs joined together to form the MLC, which currently has 29 member programs in 17 different states (Fig 1) and 2 international locations. Seven states have more than 1 member program. There are 4 member programs in California and 3 in Maryland. Florida, Georgia, Tennessee, Michigan, and Minnesota each have 2.

**Fig 1:**
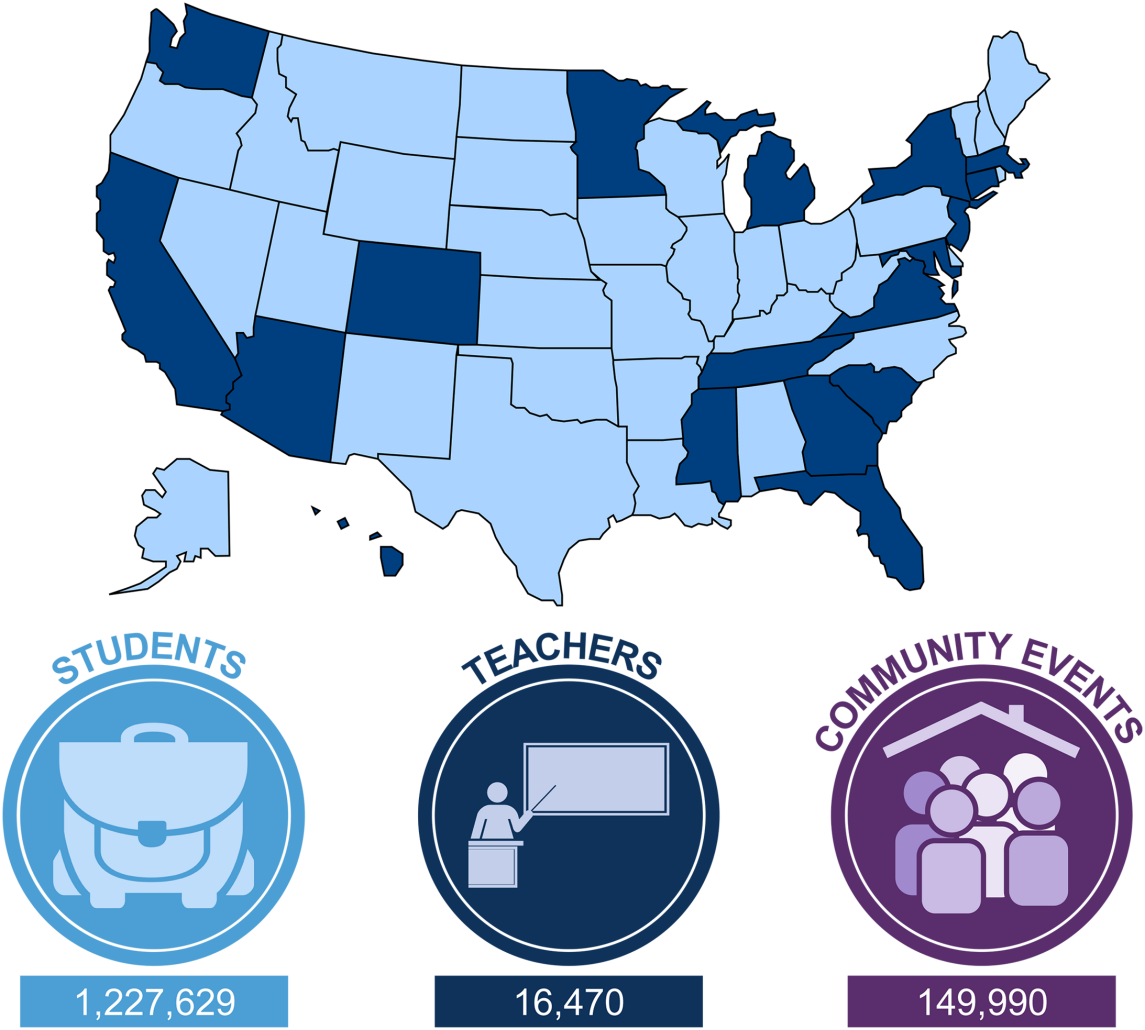
Map showing locations of US-based Mobile Lab Coalition member programs (dark blue) and total participant numbers from individual program inception through December 2015.

In an effort to learn more about the various MLC member programs, the authors recently sent an online survey to members to quantify the breadth, depth, and scope of the programming provided. Questions included information about participant numbers and demographics (if available), year of program inception, curriculum topics addressed, and the types of programming offered. The survey was also intended to collect data that could be used as a starting point for developing common measures for assessing program impact among mobile laboratory programs. The data presented here are inclusive of responding programs’ activities (*n* = 27) from individual program inception through the end of December 2015.

Collectively, the member programs of the MLC have provided hands-on science experiences to more than 1.2 million students across the US.

Many programs also offer professional development for teachers and participate in community events and science festivals (Fig 1). These data on numbers of students served highlight the critical support MLC member programs provide to formal K–12 school systems and communities that rely on innovative partnerships to support science education [26].

The survey responses revealed an impressive array of programming for elementary, middle, and high school students, covering a broad range of STEM subjects. Most programs offer instruction and hands-on investigations relevant to multiple content areas. These include programs with a focus on biology (i.e., genetics, molecular biology, and biotechnology [17 programs]), physics (13 programs), engineering (13 programs), math (11 programs), and health (10 programs). Other content areas addressed include agriculture, chemistry, Earth sciences, astrophysics, atmospheric science, bioinformatics, and simulation/gaming. Students participating in MLC member programs may have the opportunity to explore batteries, engines, electric machines, and aerodynamics; use DNA fingerprinting as a means of identifying the source of an outbreak of foodborne illness; explore the pH and nitrogen level in soil; or perform an ELISA to look for evidence of malaria. All curricula are optimized for use in a mobile laboratory and are freely shared amongst MLC members.

The majority of MLC members operate mobile laboratories. Typical vehicles used as mobile laboratories range from small vans to 45-foot RV-type vehicles and 53-foot trailers. Students are often stunned to find themselves immersed in a fully equipped science lab in the middle of their school’s parking lot (Figs 2 and 3). Some programs also have on-site laboratory facilities that students and teachers can visit (*n* = 12); eight loan scientific equipment to schools, thereby providing students access to authentic, cutting-edge equipment and resources that may not be found in classroom settings. Eighteen programs offer professional development for teachers, which can fulfill a need for high-quality, ongoing professional learning for K–12 classroom teachers [27]. The common feature among these diverse delivery methods is that all programs provide access to sophisticated laboratory tools and professional-grade research and medical equipment. Staff include scientists with backgrounds in diverse fields, graduate students, post-doctoral fellows, and educators with a science background, all of whom enthusiastically share their passion for science with students, teachers, and community members.

**Fig 2:**
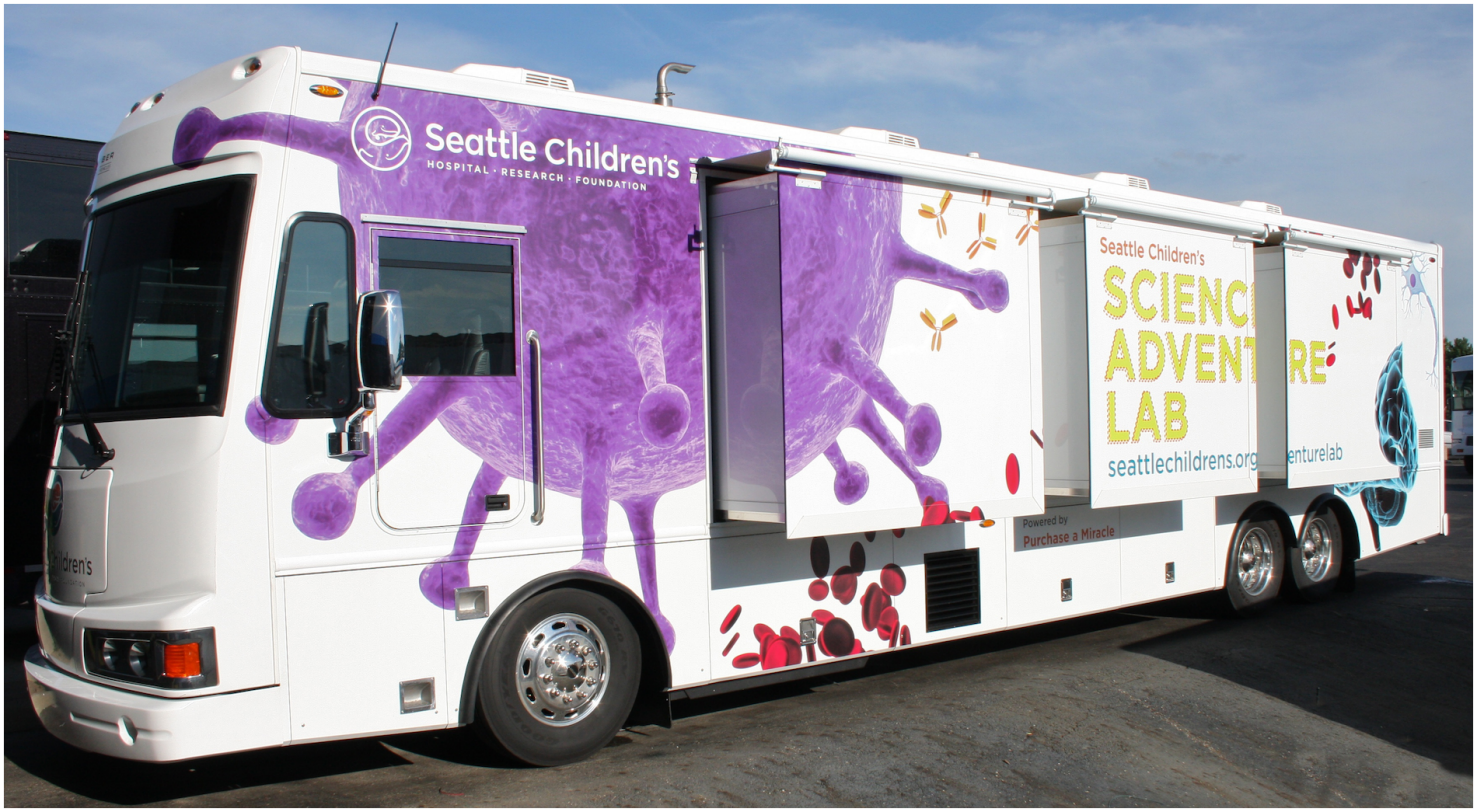
Exterior of Seattle Children’s Research Institute mobile laboratory, the Science Adventure Lab. Image credit: Farber Specialty Vehicles.

**Fig 3:**
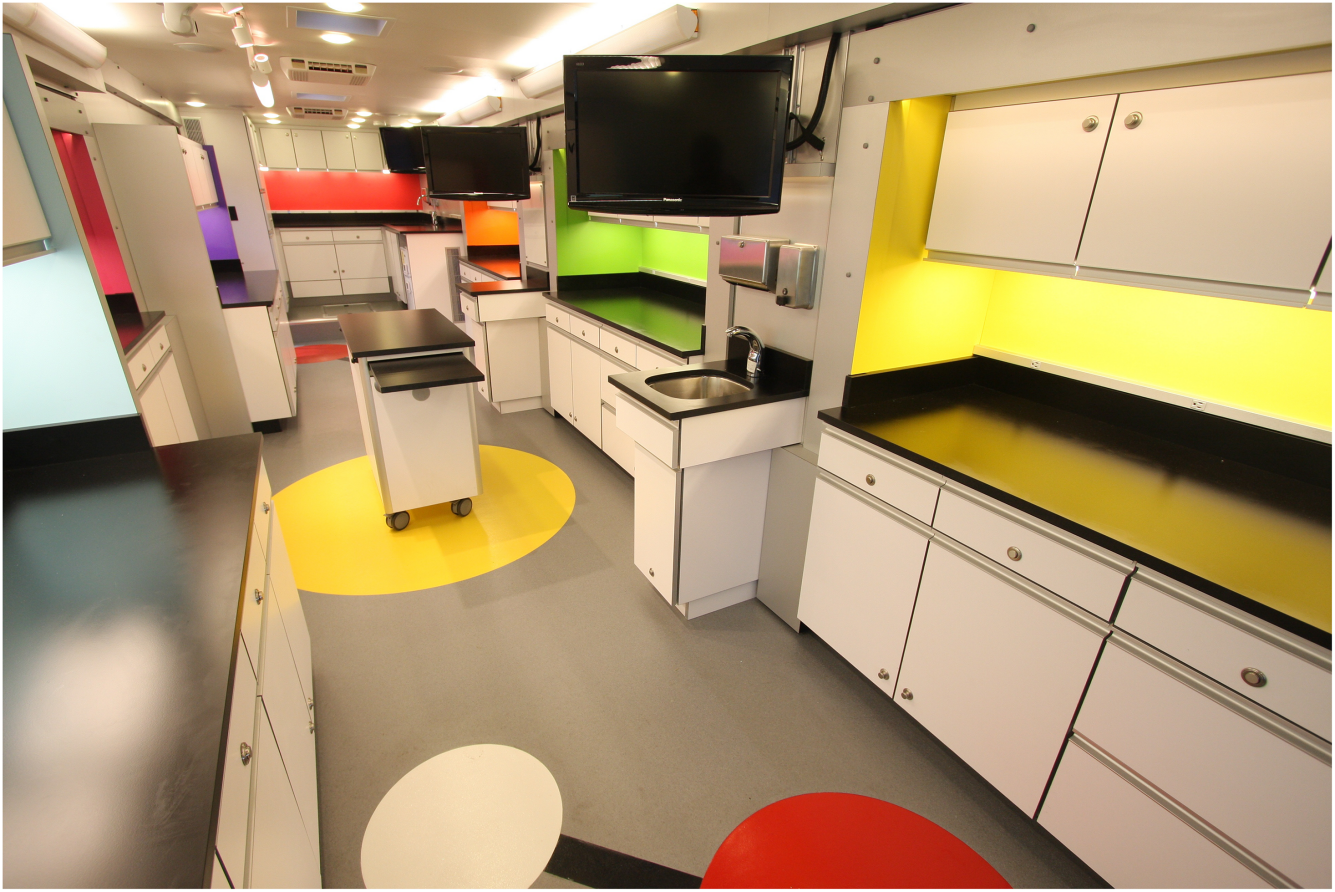
Interior of the Science Adventure Lab. Image credit: Farber Specialty Vehicles.

The ability to collect demographic and impact data varied amongst the member programs surveyed. Of the 14 programs that collect information about the schools they visit, 10 reported that >50% of their visits were to Title I schools or to schools with more than 40% of the students qualifying for free or reduced price lunch, suggesting a focus on low-resource and underserved schools. As expected, a unifying theme was a goal of improving equity of access to engaging, high-quality science education. Despite the fact that mobile laboratories have been popular since the 1990s, there is very little published literature on their impact, and many programs struggle with rigorously evaluating the impact of their program on students’ knowledge and interest in STEM. One attempt by Franzblau et al.[23] asked directors of 16 mobile laboratory programs to distribute a survey to teachers who had utilized their programs. Teachers reported that the mobile laboratory program promoted student engagement, attentiveness, and interest in additional lab-based science, as well as understanding of scientific concepts. Teachers also suggested that the experience contributed to improved performance on high-stakes tests. However, these data did not measure student learning and engagement directly but rather represented teachers’ perceptions of student learning, so the authors called for further research at the student level in order to more directly define the impact of mobile laboratory programs on student learning. However, there are several challenges to performing this type of research that we and other MLC member programs have encountered. These challenges include the difficulties in collecting meaningful data within the short duration of time students are onboard mobile laboratories, sensitivity around creating an excessive burden on teachers’ and students’ busy classroom schedules, and logistical challenges of following specific students over time to examine long-term impacts. Many programs also lack the funding for the necessary infrastructure and personnel to collect and analyze assessment data. We argue, however, that given what is known about the importance of actively engaging students in science as a way to increase learning and retention, it follows that mobile lab programs, with their focus on hands-on, inquiry-based instruction, will be effective at increasing student knowledge and interest in STEM. A long-term objective of the MLC is to begin to address the dearth of evaluation data on the effectiveness of mobile laboratory programs. To that end, the MLC is actively engaged in supporting efforts to assess program impact in mobile laboratory programs through professional development at our annual conference and via a new webinar series. These professional development opportunities are designed to help programs design and implement evaluations, find funding for such evaluation efforts, and identify and develop common measures of assessment that can be used across programs.

A major goal of the MLC is to encourage and support the development of new mobile laboratory programs. One strategy used by the MLC is to support new and emerging programs through instructor exchanges and mentoring. These activities are funded by a Science Education Partnership Award (SEPA) conference grant from the National Institutes of Health. The grant, now in its fourth year, also provides stipends that help program staff attend the annual conference, where best practices and curricula are shared and innovations in science education and assessment are discussed. Many members also frequently host site visits, field inquiries from groups interesting in starting a mobile lab program, and share their expertise and lessons learned.

The scientific community plays a significant role in supporting mobile laboratory programs. Many programs rely on scientists to serve as content experts in curriculum development, facilitators of professional development for teachers, and instructors who deliver lessons onboard their mobile labs. Scientific organizations, including businesses, nonprofits, and institutions of higher education, often provide funding for mobile lab programs.

Scientists and scientific organizations interested in becoming involved with mobile laboratory programs can contact the MLC for a list of programs in their area.

Research clearly shows that access to hands-on science activities and exposure to authentic tools are key factors in improving student achievement in science. Every student in every school deserves to experience science in an engaging way that prepares them for success and helps them become a lifelong learner and critical thinker. We, as the scientific community, must do more to support schools’ efforts—putting more mobile laboratories on the road is one way to do that.

## Supporting information

S1 Data Raw data Fig 1. (XLSX)

## Abbreviations

MLC: Mobile Lab Coalition
NAEP: National Assessment of Educational Progress
SEPA: Science Education Partnership Award
STEM: science, technology, engineering, and mathematics
