# Correction: 1.2 million kids and counting—Mobile science laboratories drive student interest in STEM

**DOI:** 10.1371/journal.pbio.1002609

**Published:** 2017-07-11

**Authors:** Amanda L. Jones, Mary K. Stapleton

Fig 1 is incorrect. Mississippi was incorrectly shaded and the authors have provided a corrected version here. The figure legend remains the same.

**Fig 1:**
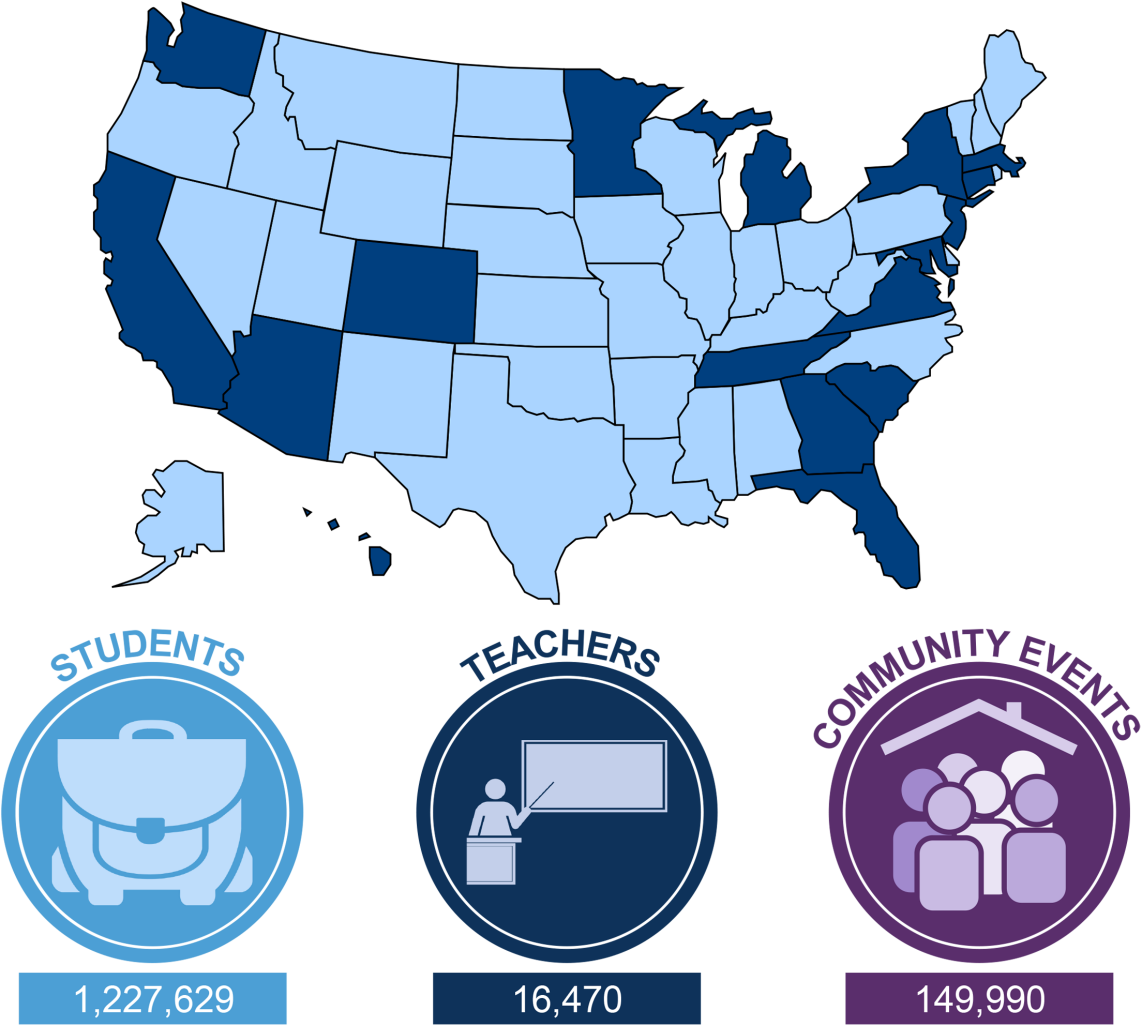
Map showing locations of US-based Mobile Lab Coalition member programs (dark blue) and total participant numbers from individual program inception through December 2015.
